# A silent culprit: Prosthetic valve endocarditis due to *Cutibacterium acnes*

**DOI:** 10.1016/j.radcr.2026.06.030

**Published:** 2026-07-02

**Authors:** Dhan Bahadur Shrestha, Ruvarashe Mupedziswa, Jeyavishnupriya Gopalkrishnan, Daniel Katz

**Affiliations:** a Division of Cardiology, Department of Internal Medicine, Bassett Medical Center, 1 Atwell Rd, Cooperstown, NY 13326, USA; b Department of Internal Medicine, Bassett Medical Center, 1 Atwell Rd, Cooperstown, NY 13326, USA

**Keywords:** *Cutibacterium acnes*, Endocarditis, Bioprosthetic valve

## Abstract

*Cutibacterium acnes* is an infrequent cause of infective endocarditis, mainly affecting patients with prosthetic valves. It’s unusual and slow-developing clinical presentation often leads to misdiagnosis as culture-negative endocarditis, causing delayed or inadequate treatment and worse outcomes. We report a man in his early fifties with a history of bicuspid aortic valve with prior surgical replacement due to group B streptococcal endocarditis, who presented to outpatient cardiology with one week of worsening shortness of breath. His ischemic evaluation was unremarkable. Transthoracic echocardiography showed a mildly reduced left ventricular ejection fraction and a leak in the bioprosthetic valve. Transesophageal echocardiography showed a suspicious mass suggestive of a vegetation and a perivalvular abscess. Initial infective workup, including blood cultures, was negative. Suspecting prosthetic valve infective endocarditis, he underwent surgical exploration and valve replacement. Cultures of surgical aortic tissue grew *C. acnes* in 1 of 3 sampled culture bottles*.* The infection was treated with antibiotics guided by infectious disease specialists. This case underscores the diagnostic and management challenges of *C. acnes* endocarditis.

## Introduction

*Cutibacterium acnes* is a common skin gram-positive commensal that causes acne. *C. acnes* accounts for 6% of prosthetic valve endocarditis [1]. Diagnosis is difficult because of its atypical presentation, with the absence of fever, elevated inflammatory markers, and a prolonged time to positive cultures [2]. These patients most commonly require surgical intervention, followed by extended antibiotic therapy, for definitive treatment. However, patients are often inadequately treated due to their atypical features, subsequently leading to increased rates of recurrent endocarditis and mortality [3].

## Case report

A 52-year-old male with a history of native bicuspid aortic valve with prior aortic valve replacement (25 mm Edwards Carpentier valve, in 2008) for group B streptococcal endocarditis presented to outpatient cardiology with 1 week of worsening shortness of breath. He also reported chest tightness the day before presentation, but denied fever, orthopnea, paroxysmal nocturnal dyspnea, edema, or constitutional symptoms. On examination in the clinic, he was noted to have a new systolic murmur and was sent to the emergency room for further assessment. Upon admission, vital signs were stable. His examination revealed a loud systolic murmur but no signs of decompensated heart failure. Laboratory workup showed elevated high-sensitivity troponins (86 pg/mL; repeat after 2 hours: 88 pg/mL) (reference normal range <20 pg/mL), brain natriuretic peptide (693 pg/ml; reference range 6-100 pg/ml), and d-dimer (1539 ng/mL; reference range 215-499 ng/mL). His electrocardiogram showed nonspecific ST-T wave changes (T-wave inversions in leads I, aVL, and V_4_-V_6_), which were new compared with the prior ECG (Fig. 1). The patient was admitted with a diagnosis of non-ST-elevation myocardial infarction (NSTEMI), and a left heart catheterization was performed, demonstrating normal coronaries (Video clip 1). Chest X-ray and computed tomography pulmonary angiogram revealed bilateral pleural effusions and patchy ground-glass opacities but no pulmonary embolism. The patient was admitted with a suspected NSTEMI and was on a heparin drip.

**Fig. 1:**
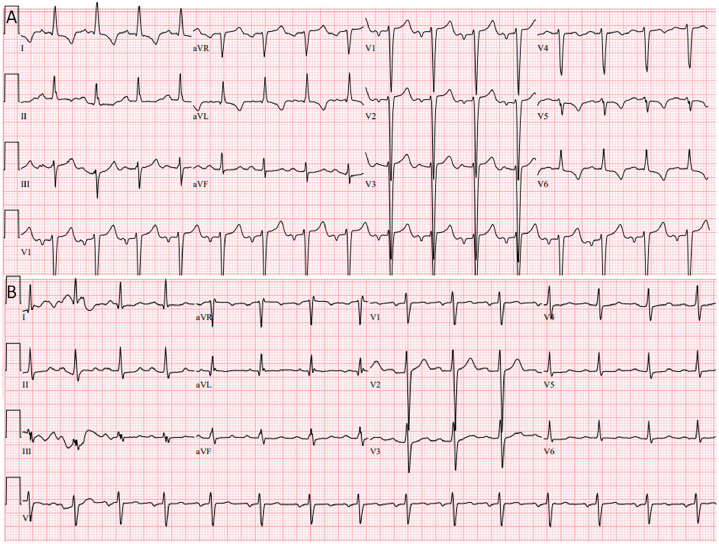
EKG (A) at the time of presentation in the hospital showing nonspecific ST-T wave changes (T wave inversions in leads I, aVL, and V_4_-V_6_), which were new compared to prior EKG; (B) Older EKG 2 years prior for comparison.

Further workup with transthoracic echocardiogram (TTE) showed left ventricular hypertrophy with mid-range left ventricular ejection fraction (45%-50%). TTE showed a new intra-valvular leak of the bioprosthetic aortic valve compared with TTE a year prior, significant worsening of prosthetic aortic valve gradients (peak/mean 55/33 mmHg [26/17 mmHg 1 year prior]; aortic valve maximum velocity of 3.7 m/s, dimensionless velocity index of 0.27, and aortic acceleration time of 130 ms), and a dilated inferior vena cava (Video clip 2). Subsequently, the TEE revealed Doppler parameters suggestive of worsening of prosthetic aortic valve stenosis and new moderate to severe eccentric aortic valve transvalvular regurgitation, with possible valve ring dehiscence/peri-valvular abscess near the left coronary artery take off in mid-esophageal aortic valve short axis window, and a 1.3 cm mass suspicious for vegetation attached to the noncoronary cusp (Video clip 3, Fig. 2). These TEE findings were also reported during intraoperative TEE at the higher center, where the patient underwent redo thoracotomy and valve replacement surgery (only report available for review).

**Fig. 2:**
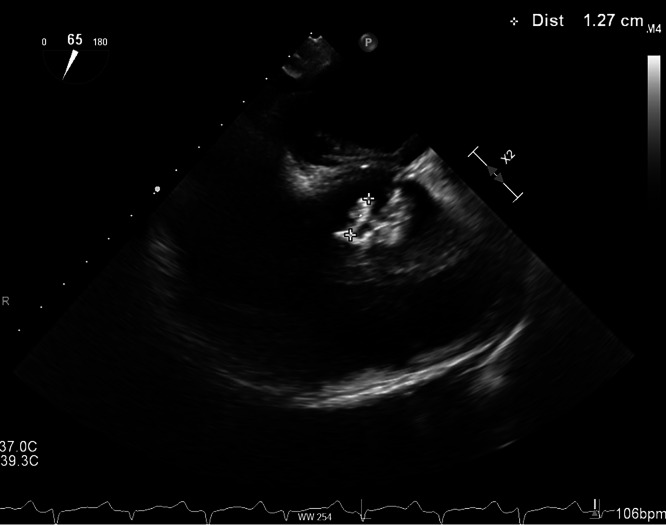
Transesophageal echocardiogram (TEE) revealed severe aortic stenosis and regurgitation, with valve ring dehiscence, and a 1.3 cm mass suspicious for a vegetation.

A normal left heart catheterization and subsequent significant transesophageal echocardiographic findings changed the course of treatment. Two sets of blood cultures and other atypical causes of infective endocarditis were ordered. The patient was started on broad-spectrum antibiotics with vancomycin and cefepime, and an infectious disease specialist was consulted. Two sets of blood cultures (1 hour apart, from different peripheral venipuncture sites), showed no growth. Extensive microbiological investigations, including *Bartonella* serology and PCR, *Cryptococcus* antigen, serum beta-d-glucan levels, *Brucella* serology, *Coxiella* serology and PCR, Q fever, urine *Legionella* antigen, and serology, all returned negative. The patient was transferred to a higher-level center for emergent surgical evaluation for redo-thoracotomy and intervention. An emergency valve replacement was planned. The valve was replaced with an Edwards *Inspiris resilia* aortic bioprosthetic valve (model 11500A), and the pathology specimen was sent for culture and analysis. Notably, intraoperative findings did not indicate vegetations or endocarditis, as assessed by infectious disease specialists and the cardiothoracic surgeon, and given that the old bioprosthetic valve was presumed to be degenerative.

A postprocedural echo showed bioprosthetic valve leaflets moving well, a well-seated valve with no perivalvular leak. Since intraoperative findings were not concerning for endocarditis and intraoperative specimen cultures were preliminary negative (48 hours) with no preceding symptoms suggestive of infection, the vegetations were thought to be solely degenerated valve and antibiotics were discontinued 2 days after surgery. The patient was discharged 3 days after valve replacement without antibiotics. His valve debris pathology only reported a degenerated valve and surrounding tissue without any pathognomic findings of infective endocarditis. After discharge, one of the 3 operative samples from the aorta grew *C. acnes.* An infectious disease specialist reached out to the patient and discussed the findings. The patient completed 6 weeks of intravenous ceftriaxone 2 g daily (Table 1). He had an excellent recovery, with symptom resolution, return to work, and normal valve function on follow-up echocardiography.

**Table 1:** Timeline of events.

Timeline	Events
16-y prior (2008)	Infective endocarditis in the setting of bicuspid aortic valve with group B *Streptococci* requiring surgical aortic valve replacement with 25 mm Edwards carpenter valve
1 y prior (2023)	Normally functioning prosthetic aortic valve with valve area of 1.6 cm^2^; pressure gradients 26/17 mmHg, dimensionless velocity index (DVI): 0.36, no aortic insufficiency
Presentation (day 0)	One week of worsening shortness of breath, new murmur, admitted for NSTEMI
Day 1	Left heart catheterization: normal coronaries; transthoracic echocardiogram: mid-range left ventricular ejection fraction (45%-50%). A new intravalvular leak of the bioprosthetic aortic valve, significant worsening of prosthetic aortic valve gradients (peak/mean 55/33 mmHg; aortic valve maximum velocity of 3.7 m/s, DVI of 0.27 and aortic acceleration time of 130 ms)
Day 2	Transesophageal echocardiogram (TEE) showed prosthetic valve stenosis and valvular regurgitation with vegetation and possible valve dehiscence/abscess. Laboratory test for infective endocarditis including 2 sets of blood culture ordered. Infectious disease (ID) consulted. Broad spectrum empiric antibiotics with vancomycin and cefepime initiated
Day 3	Two sets of blood culture collected. ID recommended to continue same antibiotics awaiting culture results and atypical causes of infective endocarditis test. Cardiothoracic surgery consulted, recommended to transfer patient to higher center given risk of redo-sternotomy. Patient accepted to higher center but didn’t got bed until next day
Day 4	Patient got bed and transferred to higher center for redo valve surgery
Day 9	Patient got valve surgery; intraoperative findings less convincing compared with prior TTE/TEE findings for infective endocarditis. Three samples of aortic fluids sent for culture. Valve debris sent for pathology
Day 11	Antibiotics stopped (post op day-1) given the intraoperative findings and another 2 sets of blood cultures collected on day 4 in the operating center also came negative for any organisms
Day 12	Discharge from hospital with outpatient appointments
Day 14	One of 3 samples of aortic fluids positive for *C. acnes*, ID service reached out to patient and discussed to start ceftriaxone for complete 6 weeks via peripherally inserted central catheter (PICC) line
Day 17	Antibiotics resumed for 6 weeks at home with nurse visiting home via PICC line
Day 59	Antibiotics completed
1 y	Patient is back to his day-to-day life and follows outpatient with his primary care provider and cardiologist

## Discussion

Prosthetic valve endocarditis (PVE) due to *C. acnes* is rare and often presents atypically, posing significant diagnostic challenges. Organisms such as *C. acnes* fall into a category of fastidious, slow-growing pathogens that frequently evade detection by standard blood culture techniques, leading to diagnostic delays and under recognition [1,4]. Unlike classic infective endocarditis, *C. acnes* PVE frequently lacks hallmark signs such as fever and elevated inflammatory markers, which contribute to diagnostic difficulty [5,6]. In this case, no systemic signs of infection were observed.

Initial investigations raised suspicion of prosthetic valve dysfunction and possible vegetation, based on echocardiographic findings. TTE raised suspicion for prosthetic valve dysfunction and potential vegetation, but the TEE was instrumental in identifying an echo-dense mass consistent with a possible vegetation. TEE’s superior sensitivity and specificity in detecting prosthetic valve complications make it the imaging modality of choice in suspected PVE, especially when TTE is inconclusive. The American Heart Association (AHA) strongly emphasizes TEE as a key tool in the diagnostic workup of blood culture-negative endocarditis and prosthetic valve infections, given its ability to detect subtle lesions [2,6]. However, in our case, intraoperative assessment at the referral center did not reveal overt signs of infection. This discrepancy highlights the often indolent and subclinical course of *C. acnes* PVE, in which vegetations may be small, sterile-appearing, or absent on macroscopic examination [5] or possibly result from partial treatment with 1 week of antibiotics before surgery.

Another diagnostic barrier may have been the delay in obtaining blood cultures after initiating broad-spectrum antibiotics, which may have contributed to initially false-negative culture results. Prior antibiotic treatment is a well-recognized cause of blood culture negativity in infective endocarditis, further complicating the diagnostic process [6]. In our case, once the patient received the TEE result, the entire endocarditis workup and antibiotics were ordered, but blood sample collection was unduly delayed until the next morning, after the patient had already received two doses of antibiotics.

The delayed growth of *C. acnes* from a single intraoperative specimen is another diagnostic difficulty. This organism is slow-growing and often considered a contaminant unless clinical suspicion remains high [1]. In our patient, there were known risk factors associated with *C. acnes* endocarditis, including having a prosthetic heart valve, male gender, and a history of previous cardiac surgery, particularly aortic valve replacement, which would make clinical suspicion high [3,5]. The positive intraoperative culture, despite the absence of intraoperative findings and negative blood cultures, prompted completion of a 6-week course of ceftriaxone, aligning with current recommendations for prosthetic valve infections [6]. There is also a possibility of contamination in the blood culture, but given the redo valve surgery and all risk factors, it was decided to pursue definitive treatment for infective endocarditis despite a single positive culture for *C. acnes,* a fastidious, slow-growing pathogen.

Our case highlights multiple challenging clinical circumstances, beginning with the fact that essential laboratory tests were delayed, and, to avoid delaying essential therapy, antibiotics were initiated early for possible infective endocarditis. Subsequently, negative blood culture results obtained after antibiotic administration, along with the possibility of fastidious organisms causing PVE, further complicated the diagnosis and treatment challenges. In the setting of antibiotic treatment for a week, the intra-operative findings didn’t match the gravity of TEE findings and given valve was already 16 years old, the intra-operative presumption was likely it was solely degenerative process but one among 3 sample of aortic fluid culture positivity on prolong culture was compelling to overturn the diagnosis of degenerative prosthetic valve disease over infective endocarditis and decision was to complete the antibiotic course covering *C. acnes*.

## Conclusion

This case reinforces the importance of maintaining a high index of suspicion for *C. acnes* in patients with prosthetic valves who present with new valve dysfunction, even in the absence of systemic inflammatory responses. Multidisciplinary collaboration among cardiology, cardiac surgery, and infectious disease specialists is crucial for navigating diagnostic uncertainty and ensuring appropriate treatment.

## Ethics approval

This is a case report; therefore, it did not require ethical approval from the ethical review committee.

## Data availability

All data related to the study are presented in the study and supplementary files.

## Patient consent

A written informed consent was obtained from the patient for the publication of this case report.

## Appendix Supplementary materials

Video 1. Coronary angiogram showing normal right and left coronary arteries. Image, application 1

Video 2. Transthoracic echocardiogram (TTE) color Doppler imaging: (A) PLAX view, (B) A3C view, (C) PSAX view, and (D) A4C view showing intravalvular leak of the bioprosthetic valve. Image, application 2

Video 3. Transesophageal echocardiogram (TEE) showing valve ring dehiscence, and a 1.3 cm mass suspicious for vegetation. Image, application 3
