# Supplementary figures and images for: A silent culprit: Prosthetic valve endocarditis due to Cutibacterium acnes
Source: Radiol Case Rep. 2026 Jul 2;21(10):4200–4. doi: 10.1016/j.radcr.2026.06.030 (PMC13352086; doi:10.1016/j.radcr.2026.06.030)

## Slide 1
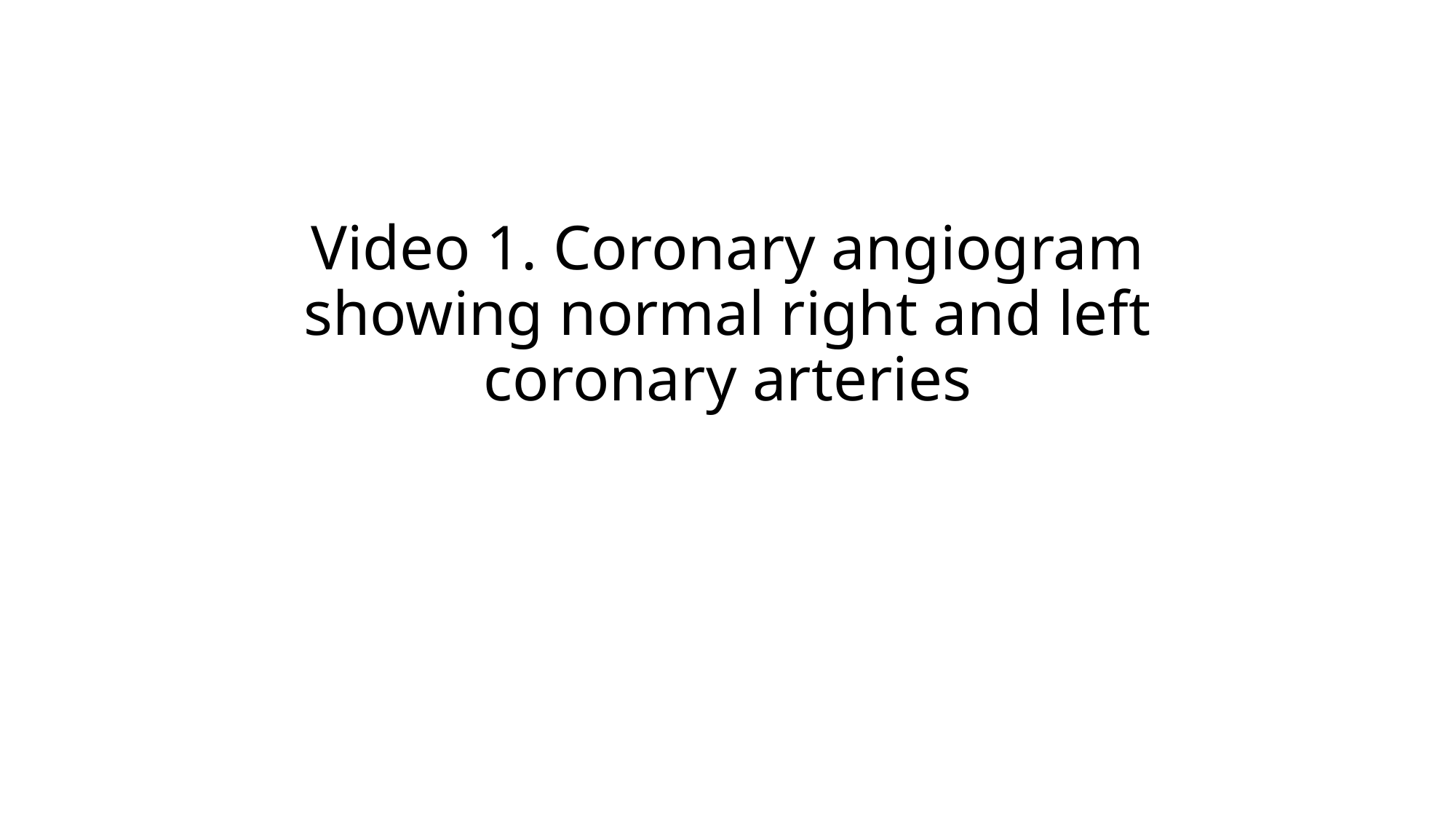

# Video 1. Coronary angiogram showing normal right and left coronary arteries

## Slide 2
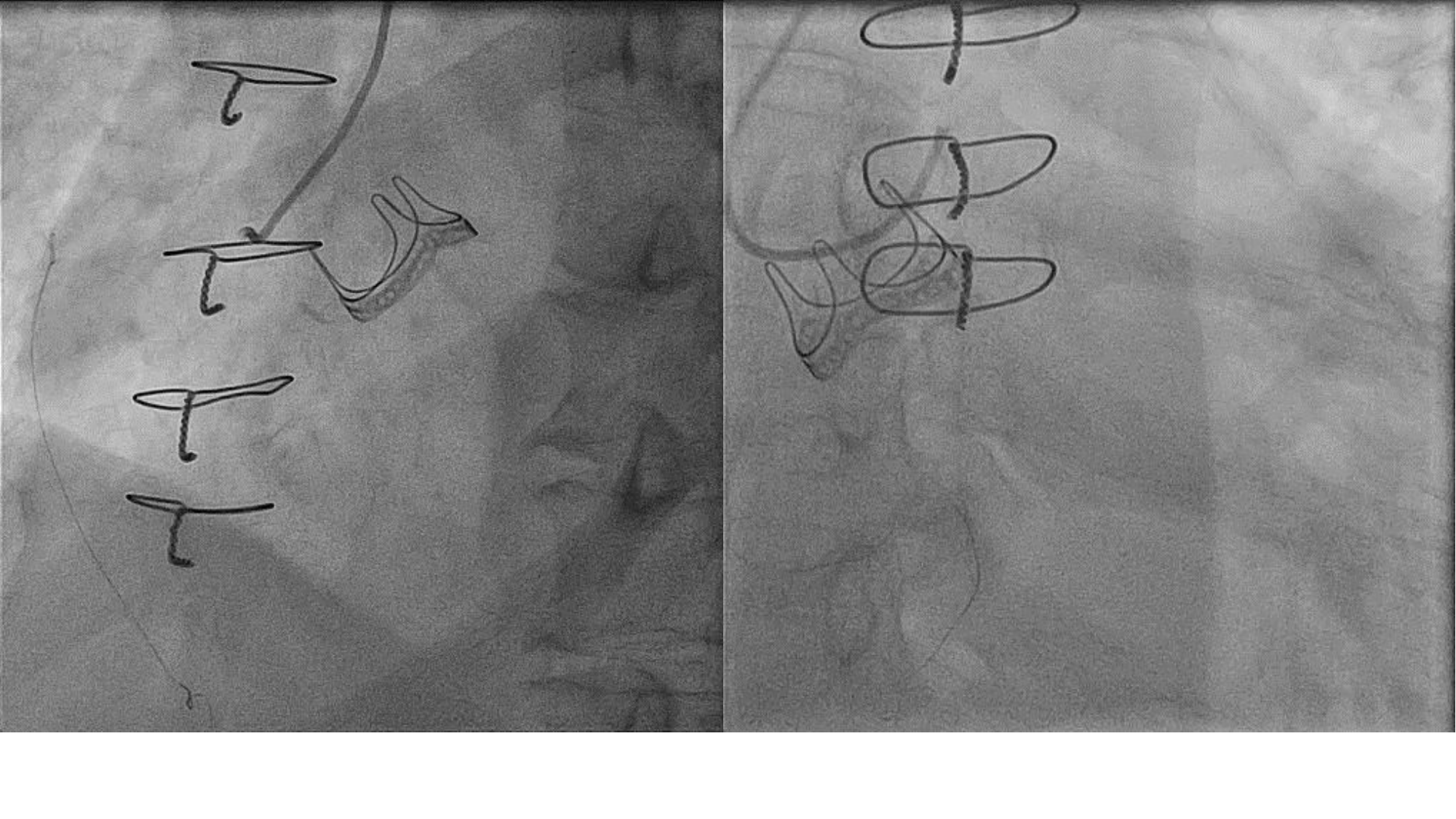

Supplement: Supplementary file 1 — Video 1. Coronary angiogram showing normal right and left coronary arteries. [file mmc1.pptx]
