# Supplementary material for: A silent culprit: Prosthetic valve endocarditis due to Cutibacterium acnes
Source: Radiol Case Rep. 2026 Jul 2;21(10):4200–4. doi: 10.1016/j.radcr.2026.06.030 (PMC13352086; doi:10.1016/j.radcr.2026.06.030)
Supplement: Supplementary file 2 — Video 2. Transthoracic echocardiogram (TTE) color Doppler imaging: (A) PLAX view, (B) A3C view, (C) PSAX view, and (D) A4C view showing intravalvular leak of the bioprosthetic valve. [file mmc2.pptx]

## Slide 1
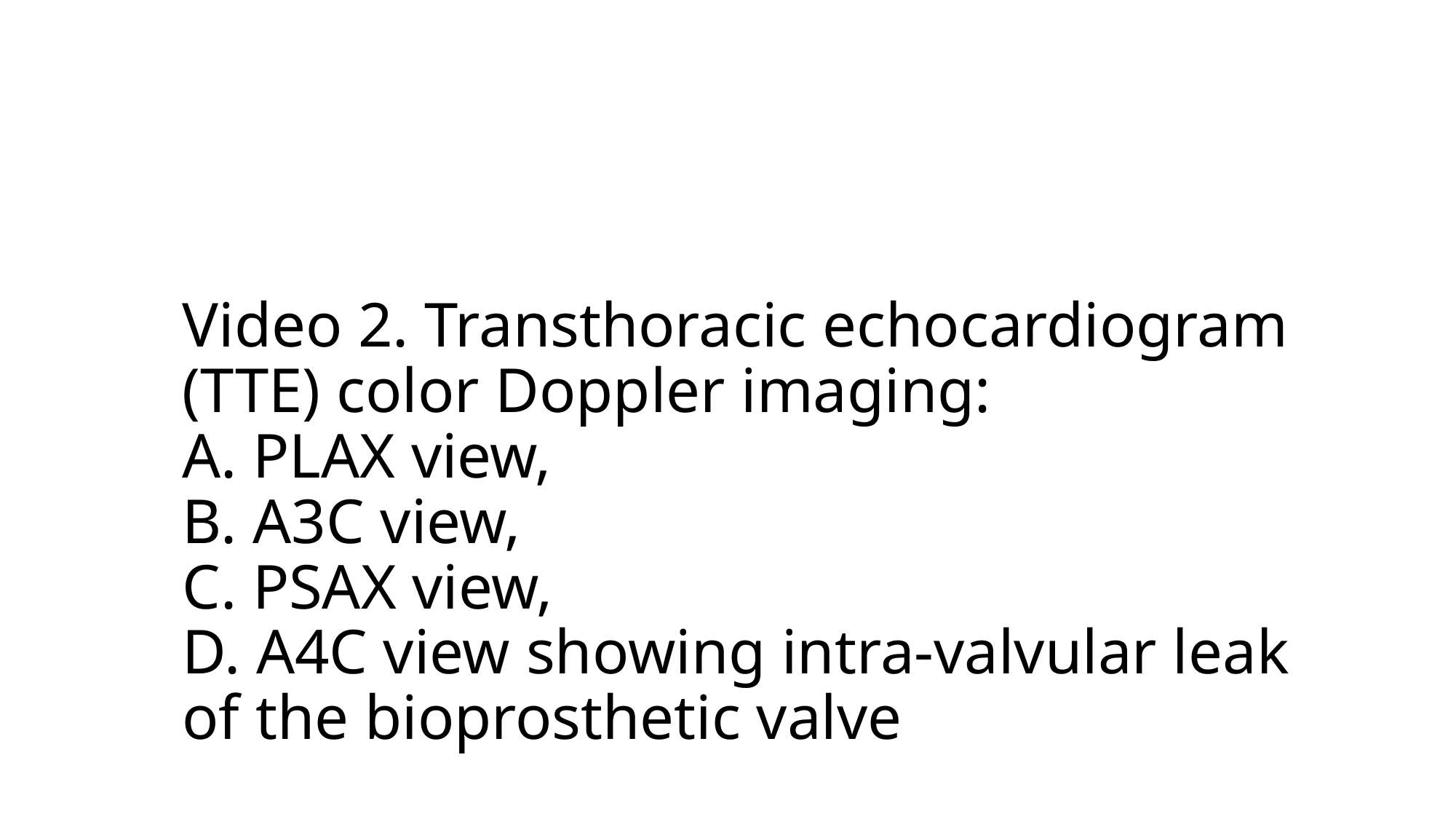

# Video 2. Transthoracic echocardiogram (TTE) color Doppler imaging: A. PLAX view, B. A3C view, C. PSAX view, D. A4C view showing intra-valvular leak of the bioprosthetic valve

## Slide 2
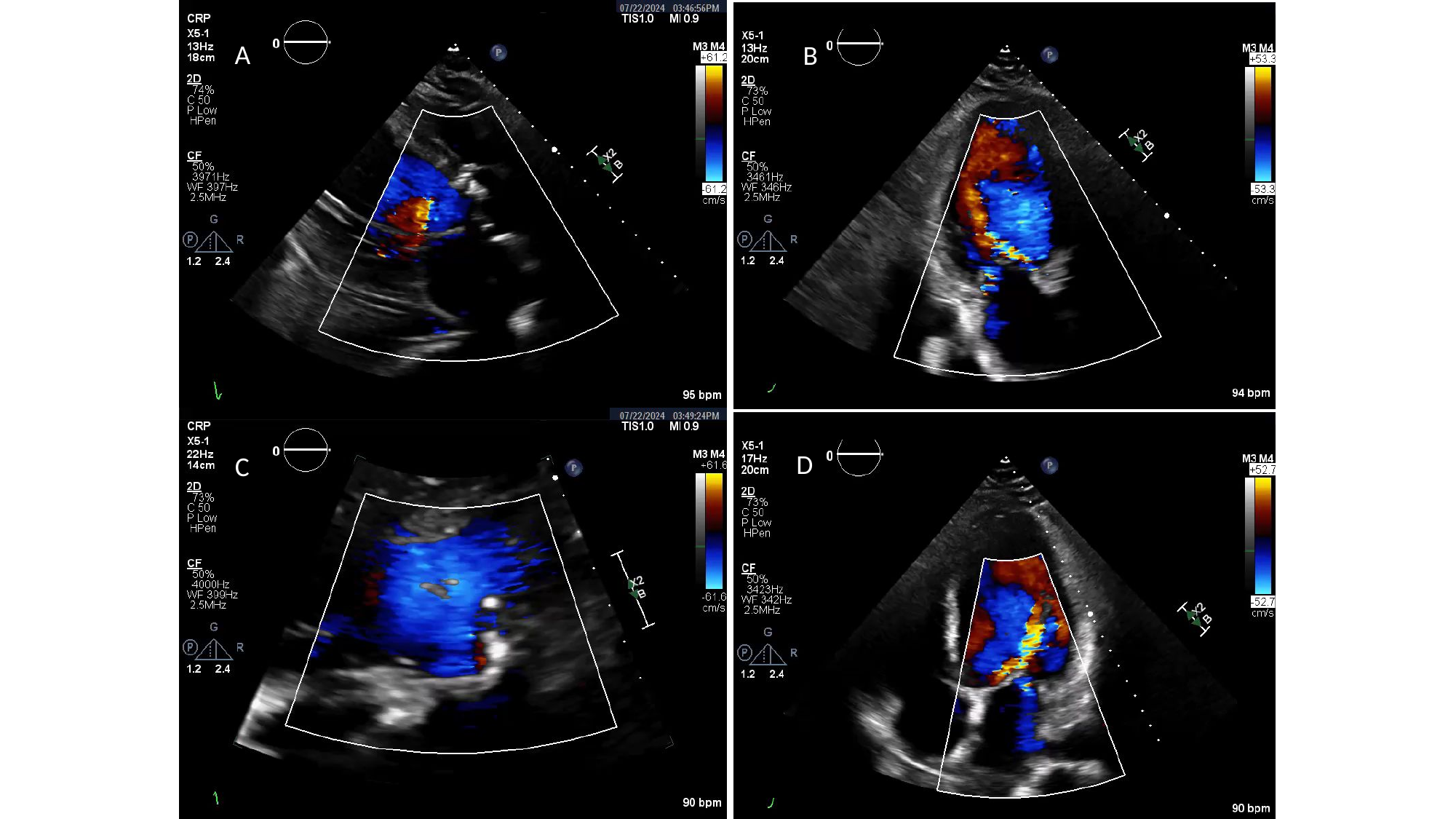

A
B
D
C
