# Supplementary material for: A silent culprit: Prosthetic valve endocarditis due to Cutibacterium acnes
Source: Radiol Case Rep. 2026 Jul 2;21(10):4200–4. doi: 10.1016/j.radcr.2026.06.030 (PMC13352086; doi:10.1016/j.radcr.2026.06.030)
Supplement: Supplementary file 3 — Video 3. Transesophageal echocardiogram (TEE) showing valve ring dehiscence, and a 1.3 cm mass suspicious for vegetation. [file mmc3.pptx]

## Slide 1
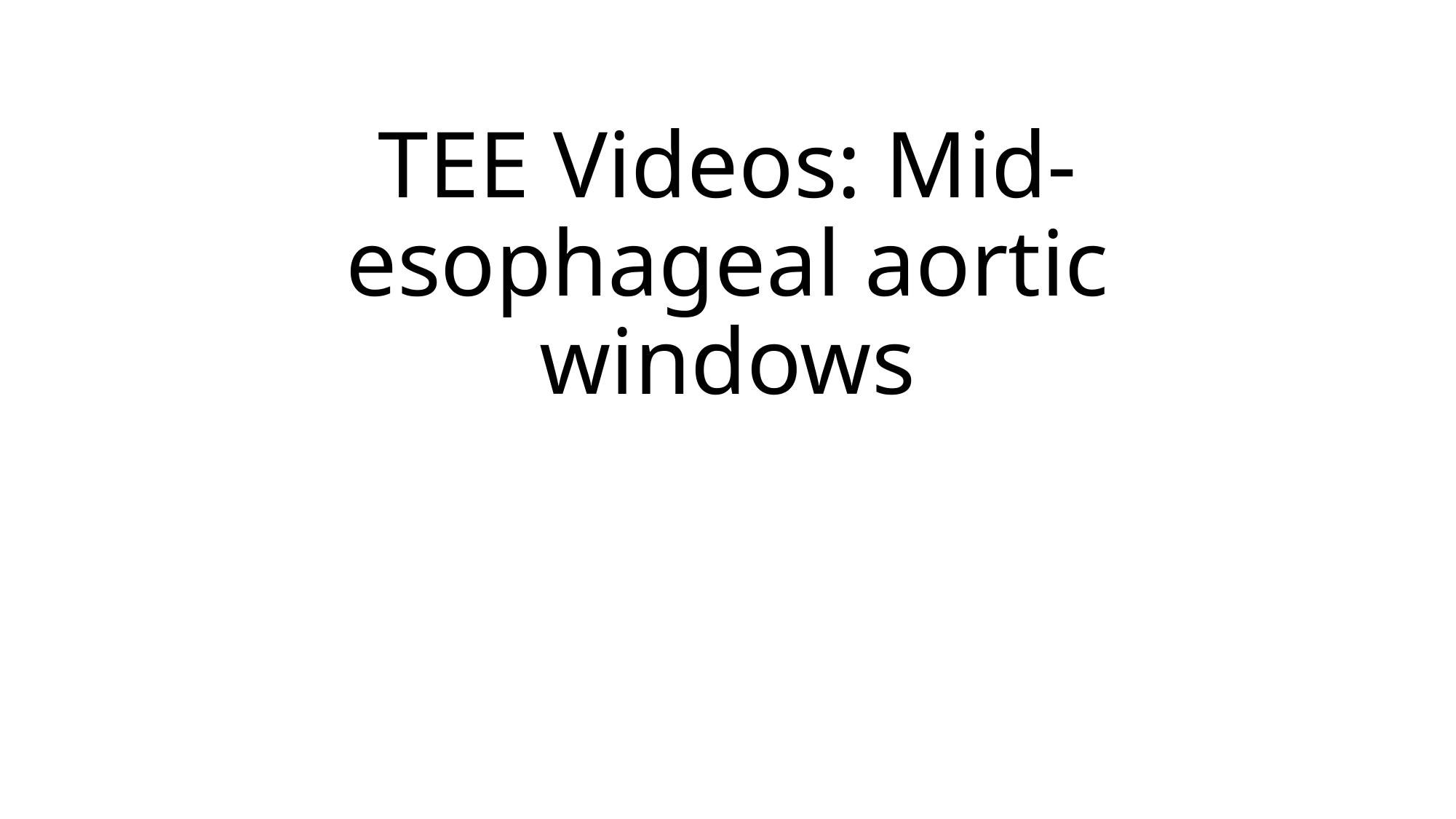

# TEE Videos: Mid-esophageal aortic windows

## Slide 2
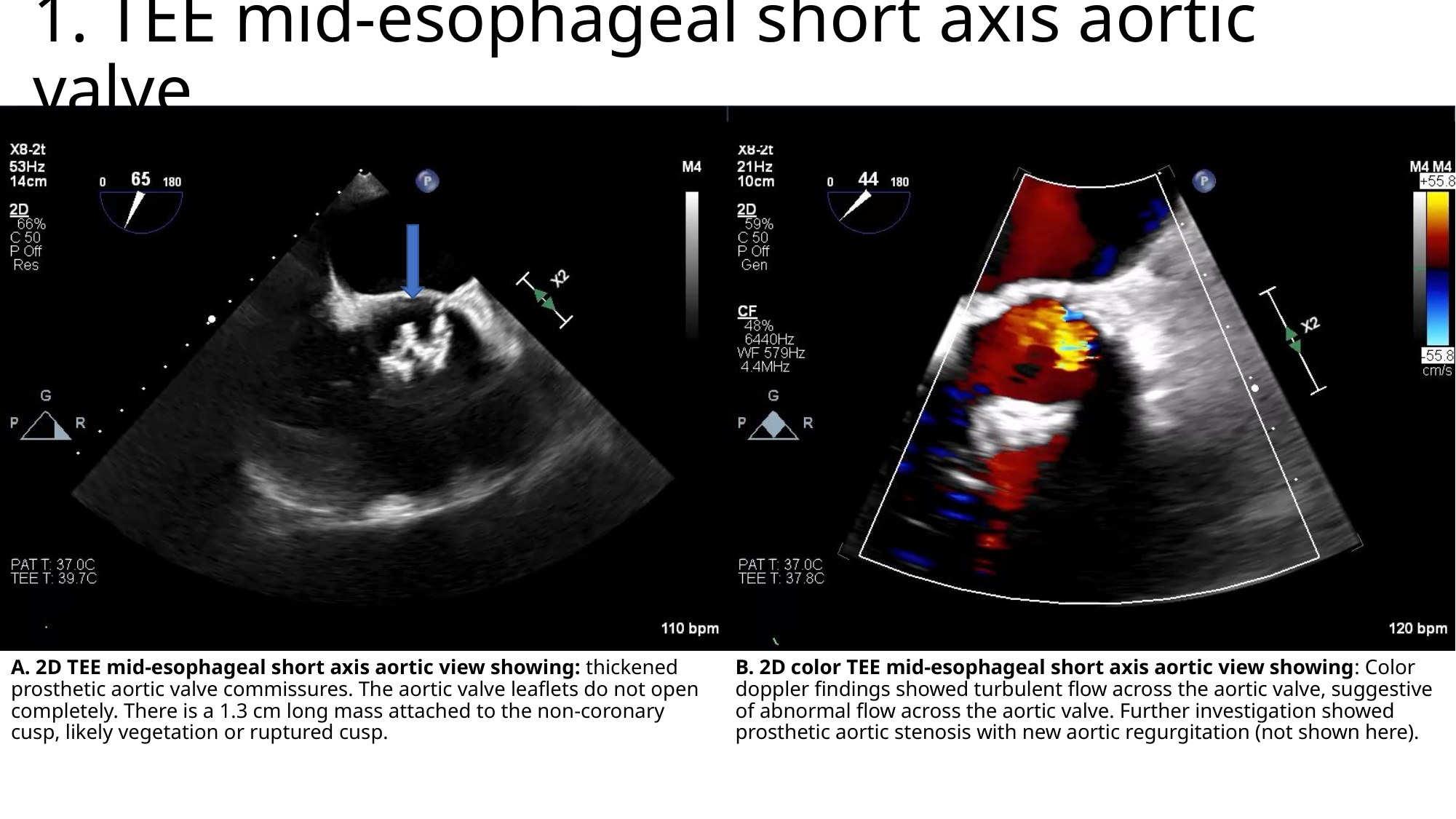

# 1. TEE mid-esophageal short axis aortic valve
A. 2D TEE mid-esophageal short axis aortic view showing: thickened prosthetic aortic valve commissures. The aortic valve leaflets do not open completely. There is a 1.3 cm long mass attached to the non-coronary cusp, likely vegetation or ruptured cusp.
B. 2D color TEE mid-esophageal short axis aortic view showing: Color doppler findings showed turbulent flow across the aortic valve, suggestive of abnormal flow across the aortic valve. Further investigation showed prosthetic aortic stenosis with new aortic regurgitation (not shown here).

## Slide 3
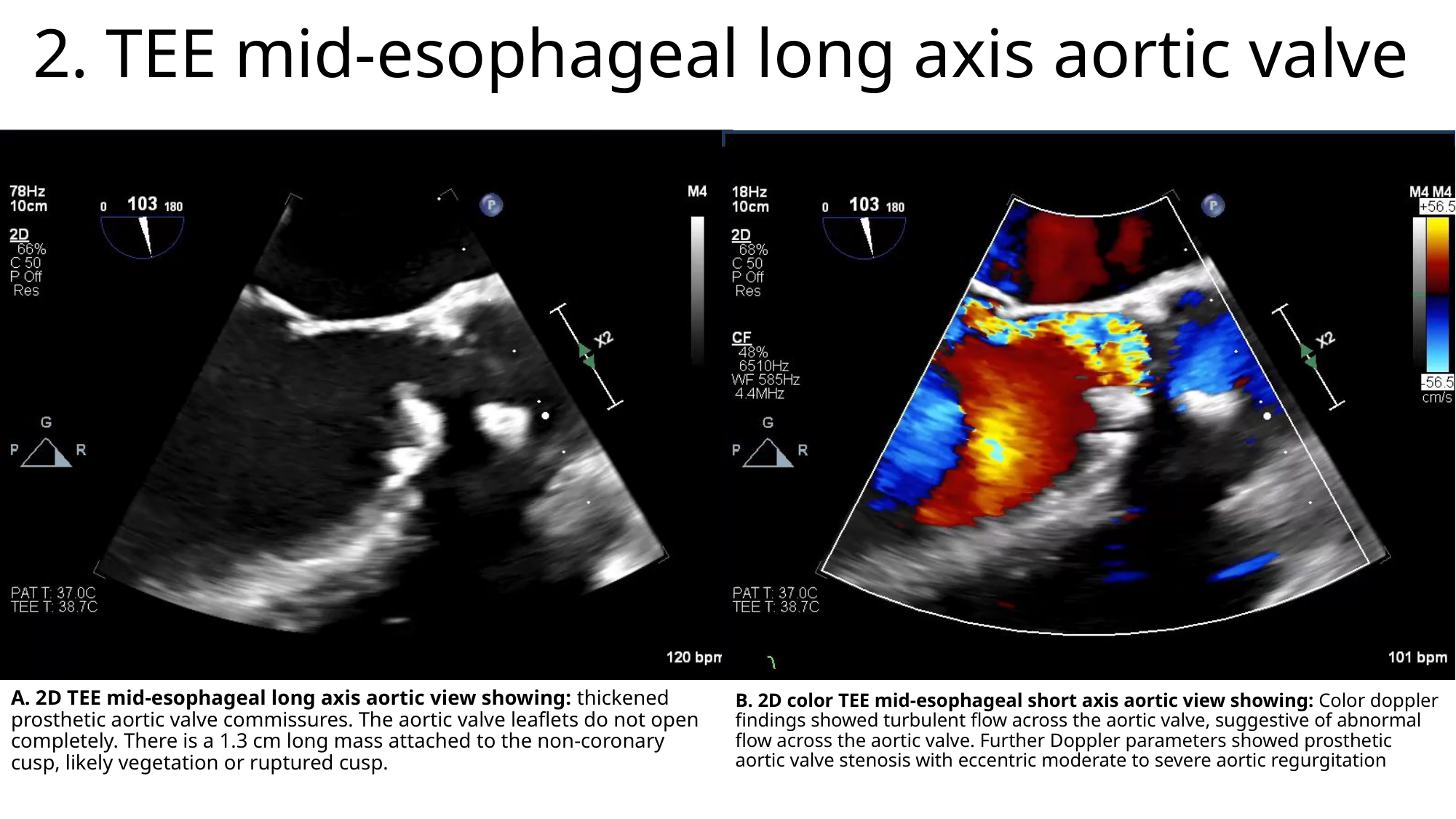

# 2. TEE mid-esophageal long axis aortic valve
A. 2D TEE mid-esophageal long axis aortic view showing: thickened prosthetic aortic valve commissures. The aortic valve leaflets do not open completely. There is a 1.3 cm long mass attached to the non-coronary cusp, likely vegetation or ruptured cusp.
B. 2D color TEE mid-esophageal short axis aortic view showing: Color doppler findings showed turbulent flow across the aortic valve, suggestive of abnormal flow across the aortic valve. Further Doppler parameters showed prosthetic aortic valve stenosis with eccentric moderate to severe aortic regurgitation
